# Supplementary material for: Emergent topological polarization textures in relaxor ferroelectrics
Source: Nat Commun. 2025 Aug 13;16:7531. doi: 10.1038/s41467-025-62658-1 (PMC12350780; doi:10.1038/s41467-025-62658-1)
Supplement: Supplementary file 1 — Supplementary information [file 41467_2025_62658_MOESM1_ESM.pdf]

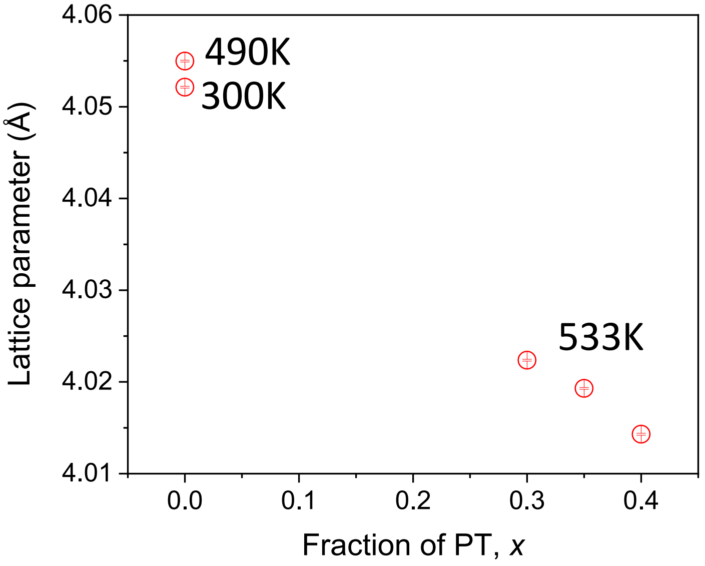

Fig. S1**: Lattice parameter in PMN-PT.** Dependence of the cubic lattice parameter on the PT content, *x*. For PMN, the values are shown for 300 K and 490 K. Values for *x*=0.3, 0.35, and 0.40 correspond to T=533 K. Error bars, which fit within the symbol size, reflect single standard deviations estimated by TOPAS.

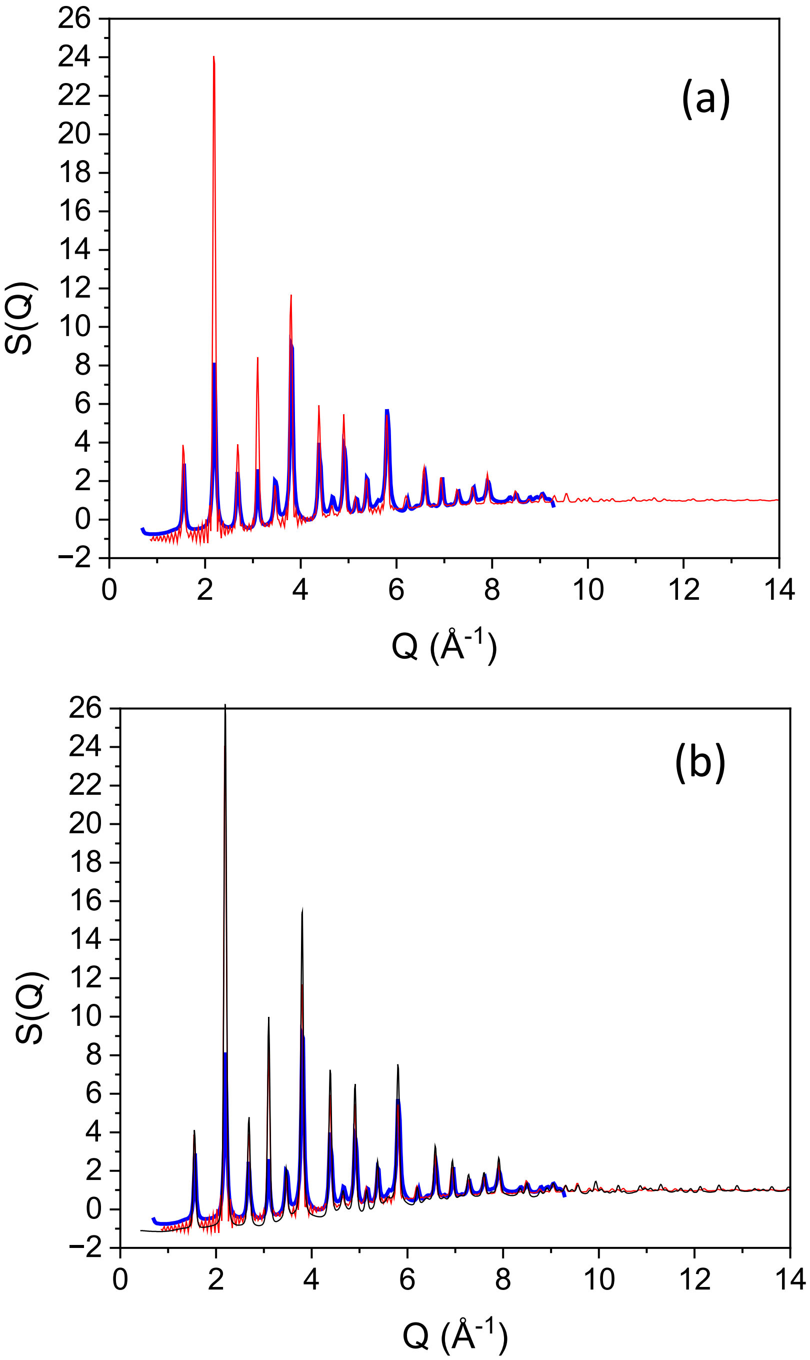

Fig. S2: **Placing diffuse scattering on the absolute scale**. (a) (blue) X-ray S(Q) in PMN, which was obtained by spherically averaging the experimental 3D distribution of the diffuse-scattering intensity for PMN. The resulting intensity trace was properly normalized and rescaled for its baseline to match that in the X-ray powder S(Q) (red) calculated directly for an atomic configuration representing PMN, where atoms were displaced randomly according to their ADP tensors obtained from Rietveld refinements. The scale factor determined from this procedure was applied to the experimental 3D diffuse scattering dataset to bring it to the absolute scale. (b) Same as (a) but with an additional trace (black) representing the experimental X-ray powder S(Q).

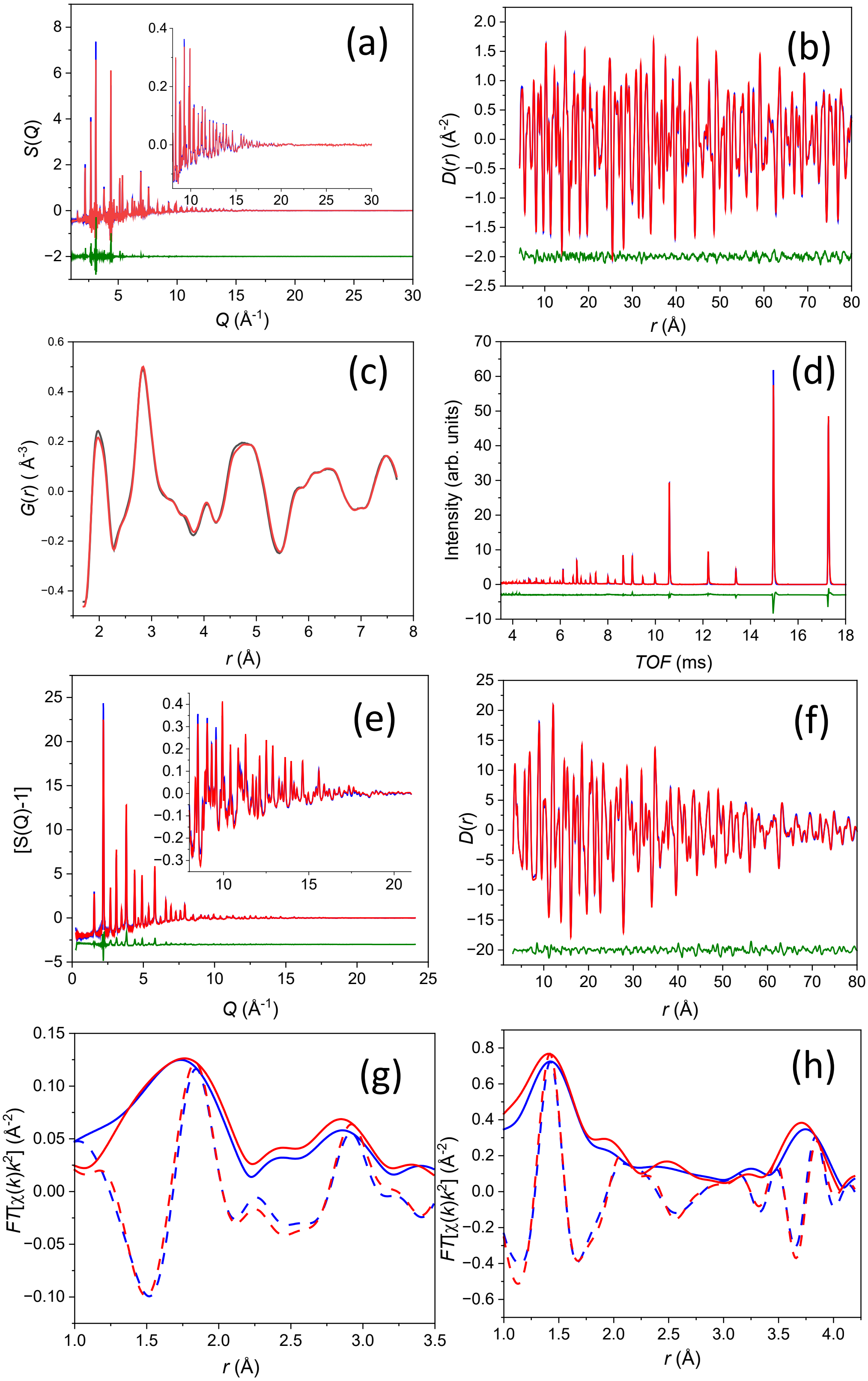

Fig. S3: **Fitting results for PMN**. Experimental (blue) and calculated (red) powder data for PMN at 300 K. The calculated signals are for a configuration refined using RMCProfile. (a) Neutron scattering function S(*Q*); (b) neutron PDF (c) local range of the neutron PDF, (d) neutron Bragg profile; (e) X-ray [S(*Q*)-1] with the inset showing a magnified view of the high-*Q* range; (f) X-ray PDF, (G) Pb EXAFS, and (h) Nb EXAFS. In (a) through (f), green lines represent the difference. In (g) and (h), solid and dashed lines correspond to the magnitude and imaginary part of the Fourier transform (FT), respectively. The *k*-ranges used in the FT are from k=2.16 Å^{-1} to 10.05 Å^{-1} for Pb and from k=3.5 Å^{-1} to k=12.1 Å^{-1} for Nb.

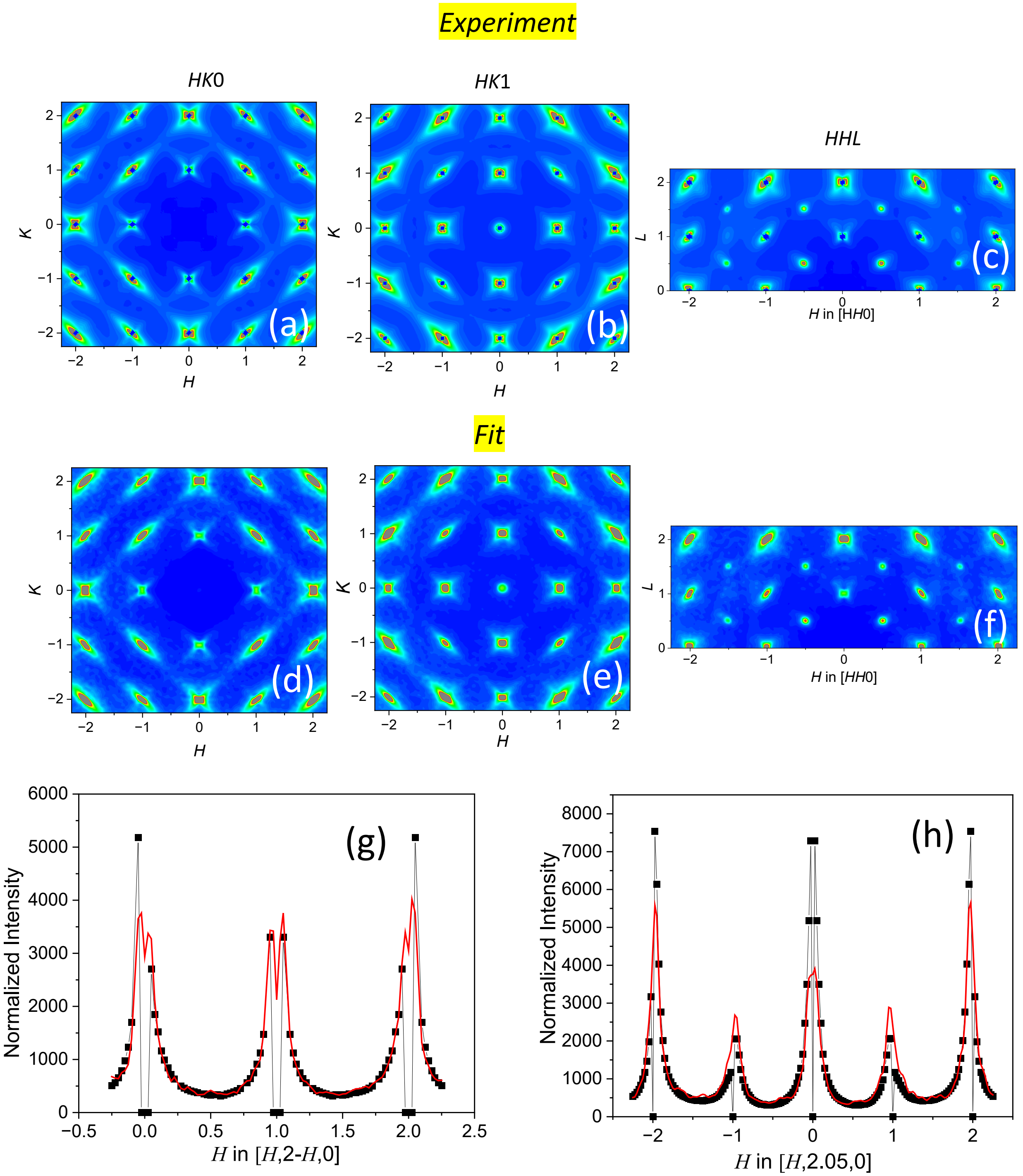

Fig. S4: **Fitting results for PMN at 300 K.** *HK*0, *HK*1, and *HHL* sections of the 3D X-ray diffuse intensity distribution in PMN at 300 K fitted in RMCProfile together with the powder data, which are shown in Fig. S3. (a-c) – experiment; (d-f)– fit; (g, h) – selected traces comparing the experimental (black) and fitted (red) diffuse intensity. In the experimental data, the pixels around the saturated reflections were excluded from the fit. The procedure for calculating the diffuse scattering in RMCProfile implies that the *calculated* intensity at the exact positions of Bragg peaks is identically equal to zero. The non-zero intensity at these locations is a result of the smoothing applied to the calculated signal. This intensity is unreliable and should be ignored.

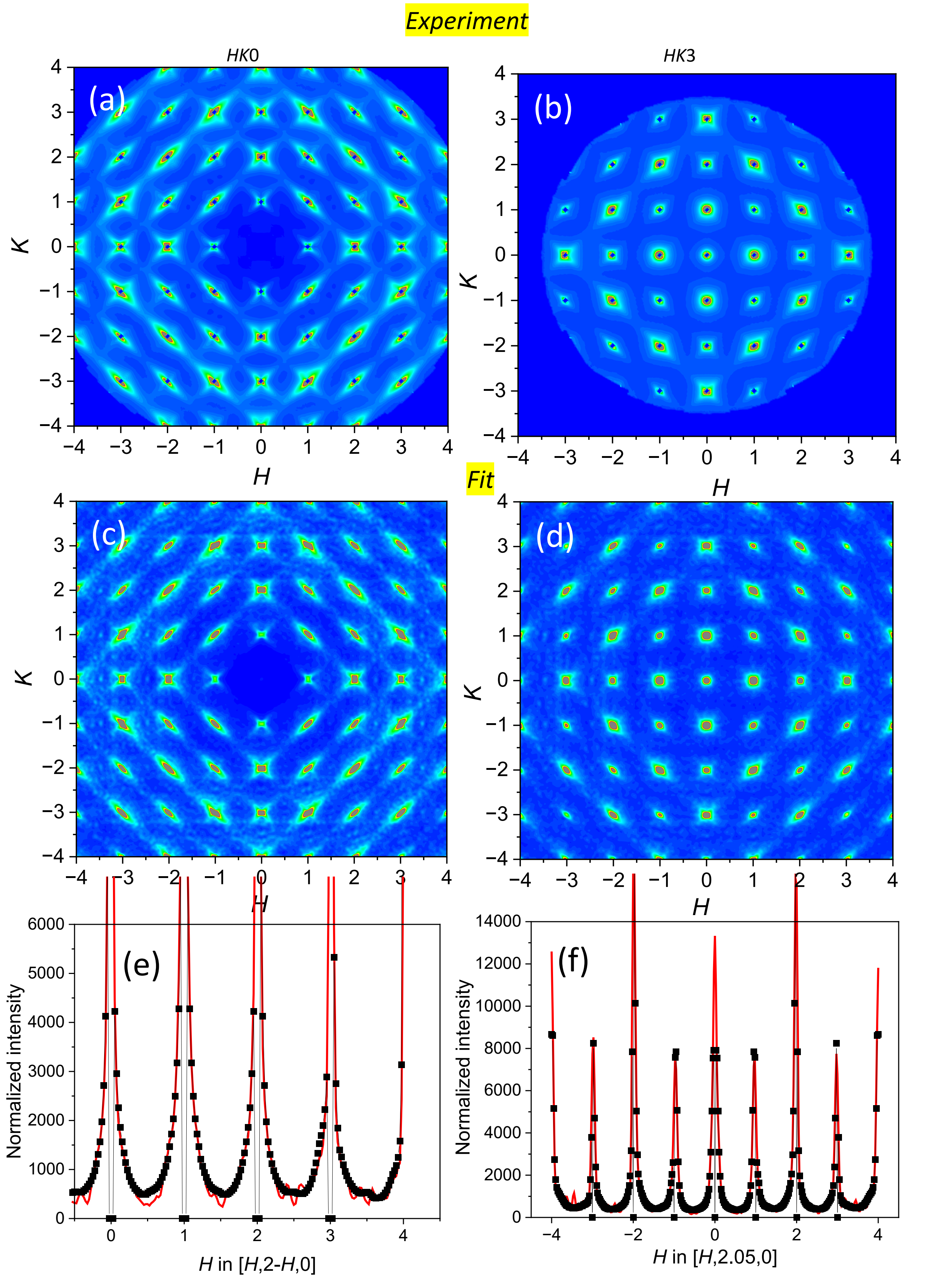

Fig. S5: **Diffuse scattering in PMN at 300 K over the wider *HKL* range**. Sections (a, c) *HK0* and (b, d) *HK*3 shown out to *H*=±4, *K*=±4. While the fit was performed only up to *H*=±2, *K*=±2, the resulting configuration describes the experimental intensity satisfactorily over a much broader range, as evident from the comparison of the intensity traces in (e) and (f).

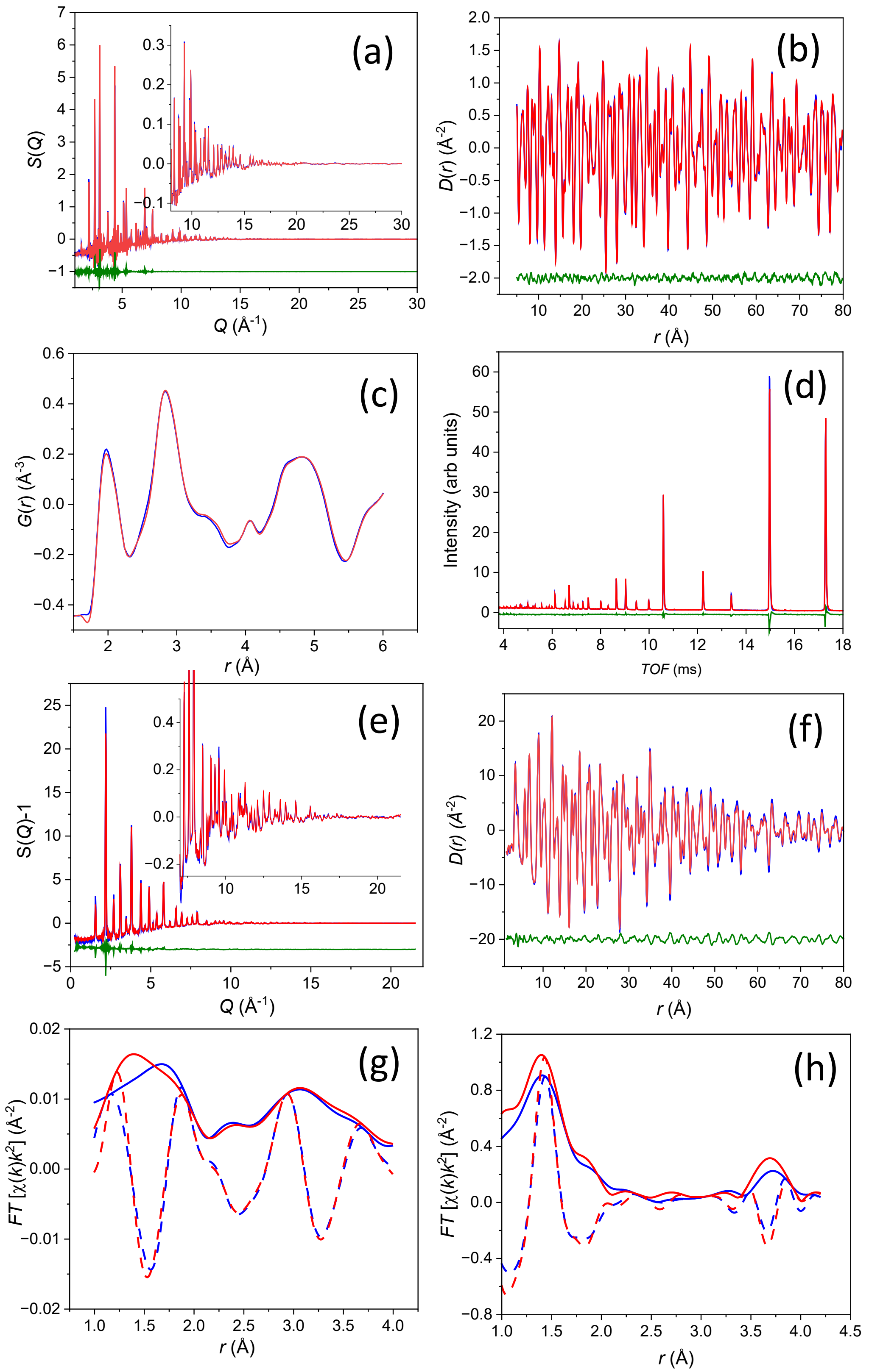

Fig. S6: **Fitting results for PMN at 490K**. Experimental (blue) and calculated (red) powder data for PMN at 490 K. The calculated signals are for a configuration refined using RMCProfile. (a) Neutron scattering function S(Q); (b) neutron PDF (c) local range of the neutron PDF, (d) neutron Bragg profile; (e) X-ray [S(Q)-1] with the inset showing a magnified view of the high-Q range; (f) X-ray PDF, (g) Pb EXAFS, and (h) Nb EXAFS. In (a) through (f), green lines represent the difference. In (g) and (h), solid and dashed lines correspond to the magnitude and imaginary part of the Fourier transform (FT), respectively. The k-ranges used in the FT are from 3 Å^{-1} to 10.7 Å^{-1} for Pb and from 2.1 Å^{-1} to 12.1 Å^{-1} for Nb.

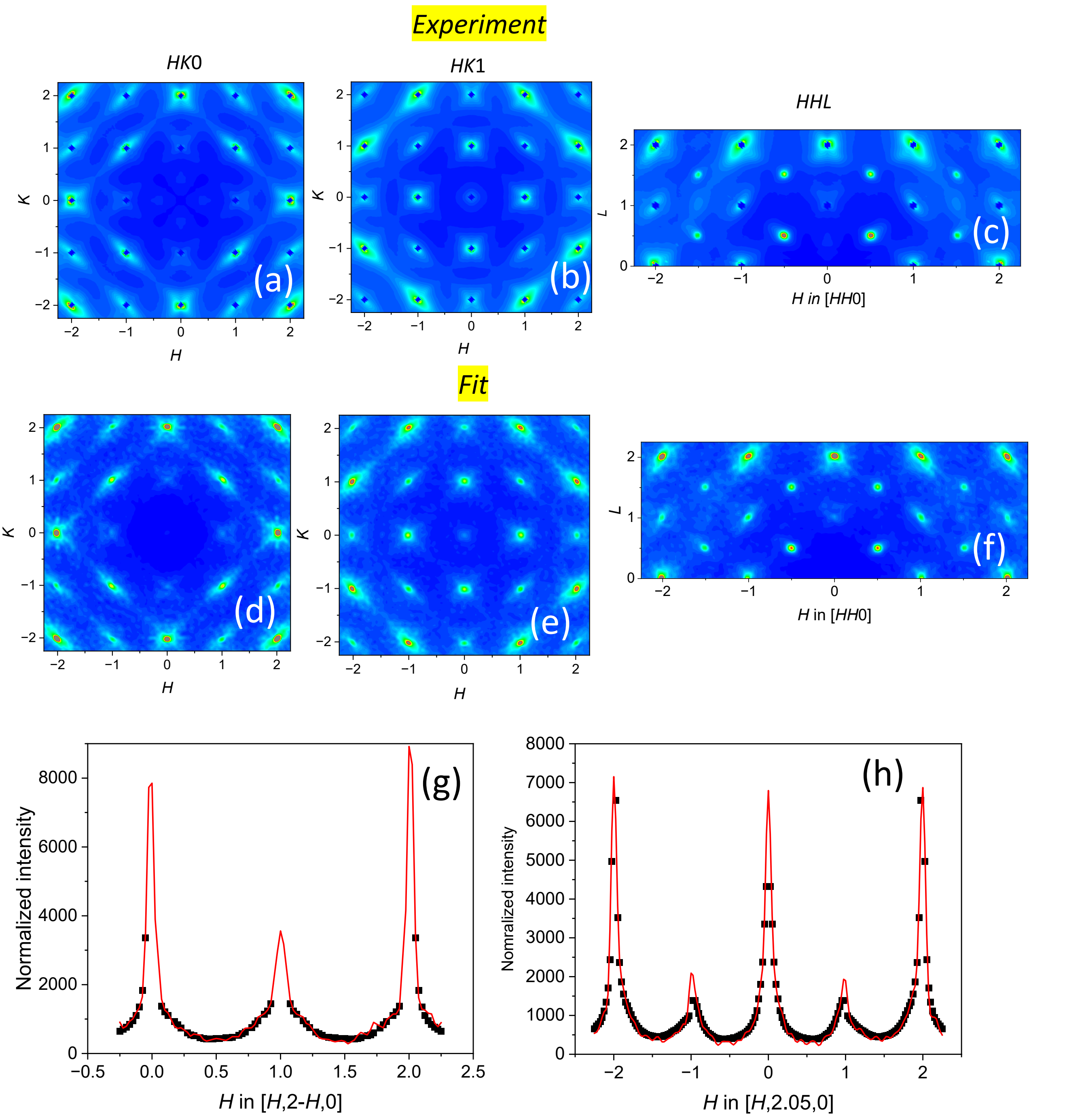

Fig. S7: **Fitting results for PMN at 490 K**. *HK*0, *HK*1, and *HHL* sections of the 3D X-ray diffuse intensity distribution in PMN at 490 K fitted in RMCProfile together with the powder data shown in Fig. S6. (a-c) – experiment; (d-f) – fit; (g), (h) – selected traces comparing the experimental (black) and fitted (red) diffuse intensity. In the experimental data, the pixels around the saturated reflections were excluded from the fit. The procedure for calculating the diffuse scattering in RMCProfile implies that the *calculated* intensity at the exact positions of Bragg peaks is identically equal to zero. The non-zero intensity at these locations is a result of the smoothing applied to the calculated signal. This intensity is unreliable and should be ignored.

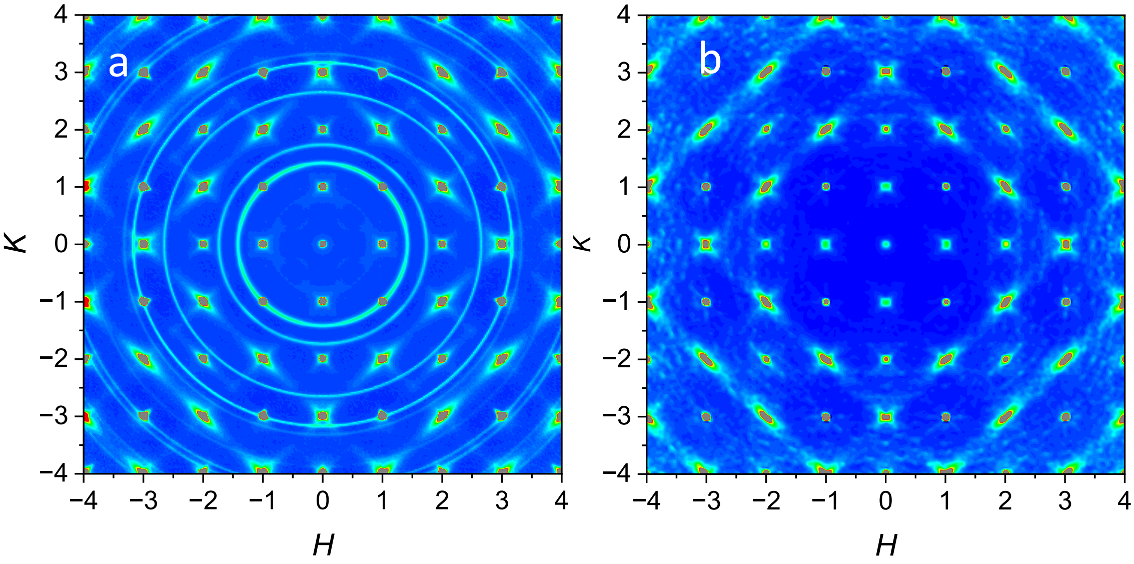

Fig. S8: **Neutron diffuse scattering in PMN**. (a) *HK*1 section of the 3D neutron diffuse intensity distribution in PMN at 300 K (processed with the correlation chopper off). The ***Q***-vectors for Bragg reflections in this section are directed out of the plane, and therefore, the streaking caused by the instrumental profile function is not visible. The powder diffraction rings arise from the aluminum shielding. These neutron data were not included in the fit. Nevertheless, the intensity distribution calculated for the refined configuration (b) reproduces the characteristic features of the experimental dataset.

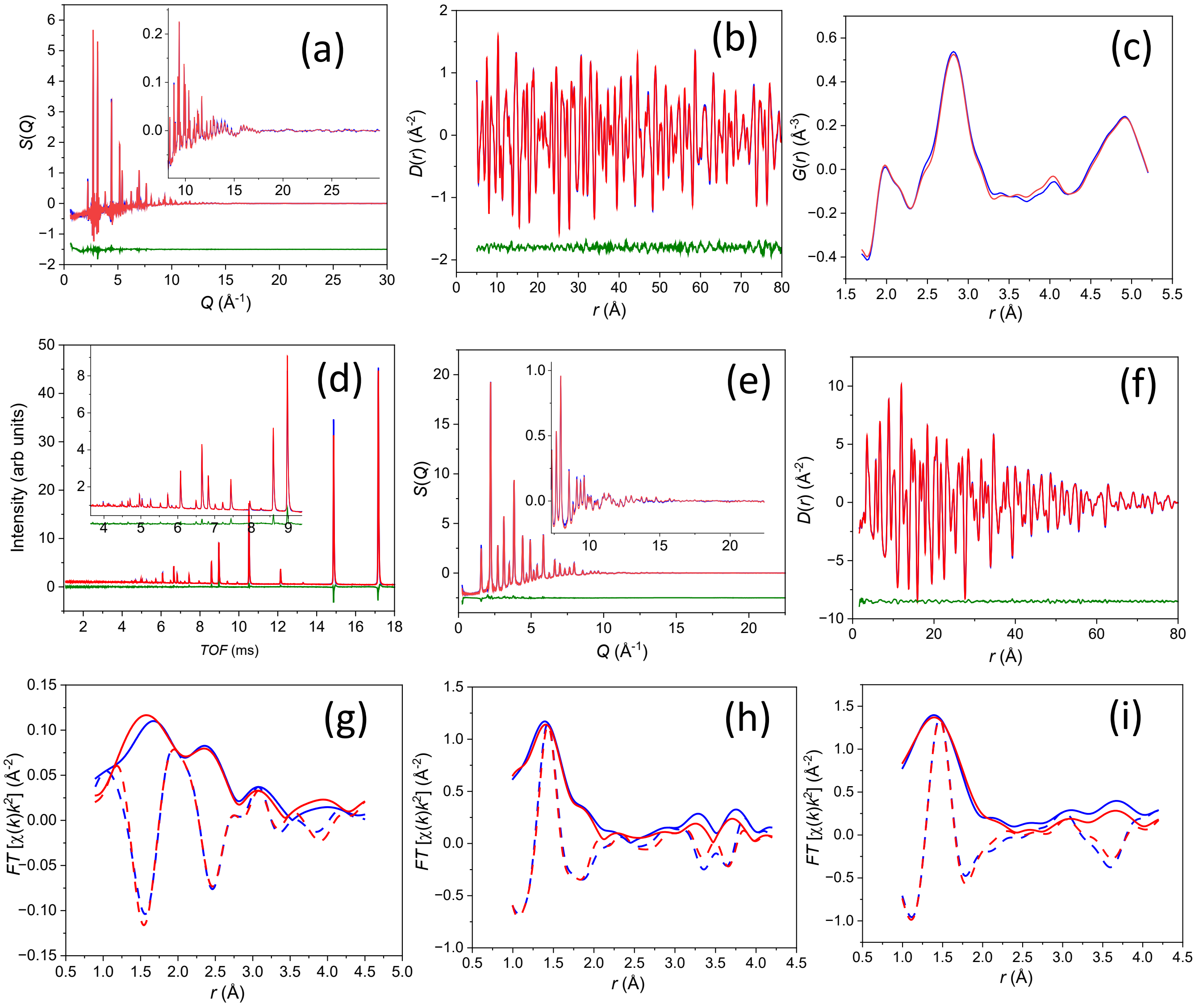

Fig. S9: **Fitting results for PMN-30PT**. Experimental (blue) and calculated (red) powder data for PMN-30PT at 533 K. The calculated signals are for a configuration refined using RMCProfile. (a) Neutron scattering function S(Q); (b) neutron PDF (c) local range of the neutron PDF, (d) neutron Bragg profile; (e) X-ray scattering function with the inset showing a magnified view of the high-Q range; (f) X-ray PDF, (g) Pb EXAFS, (h) Nb EXAFS, (i) Ti EXAFS. In (a) through (f), green lines represent the difference. In (g), (h), and (i) solid and dashed lines correspond to the magnitude and imaginary part of the Fourier transform (FT), respectively. The *k*-ranges used in the FT are 2.3 Å^{-1} to 9 Å^{-1} for Pb, 2.1 Å^{-1} to 12 $^{-1}$ Å^{-1} for Nb, and 2.2 Å^{-1} to 8.3 Å^{-1} for Ti.

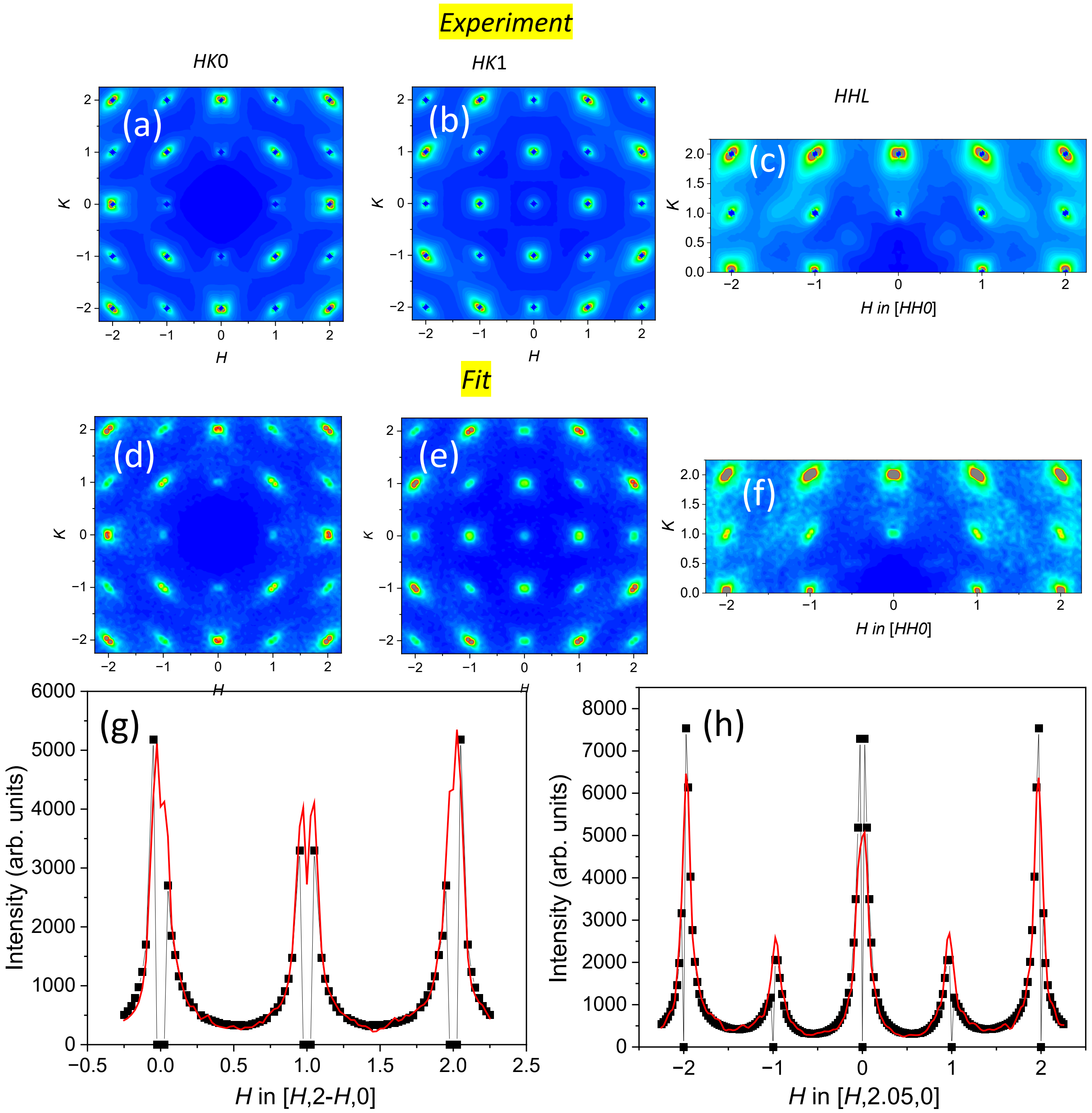

Fig. S10**: Fitting results for PMN-30PT**. *HK*0, *HK*1, and *HHL* sections of the 3D X-ray diffuse intensity distribution in PMN-30PT at 533 K fitted in RMCProfile together with the powder data shown in Fig. S9. Top row – experiment; middle row – fit; bottom row – selected traces comparing the experimental (black) and fitted (red) diffuse intensity. In the experimental data, the pixels around the saturated reflections were excluded from the fit. The procedure for calculating the diffuse scattering in RMCProfile implies that the *calculated* intensity at the exact positions of Bragg peaks is identically equal to zero. The non-zero intensity at these locations is a result of the smoothing applied to the calculated signal. This intensity is unreliable and should be ignored.

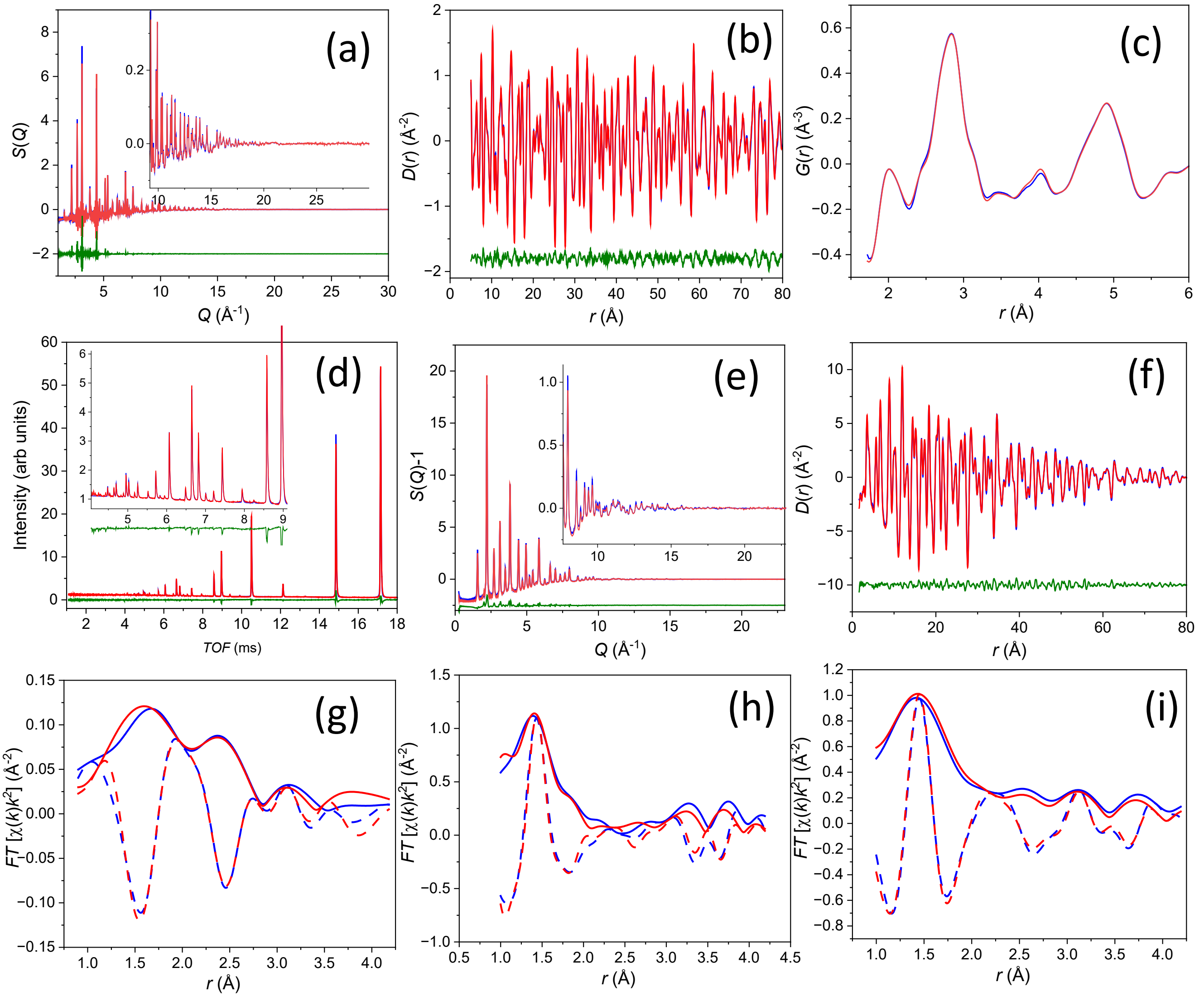

Fig. S11: **Fitting results for PMN-35PT**. Experimental (blue) and calculated (red) powder data for PMN-35PT at 533 K. The calculated signals are for a configuration refined using RMCProfile. (a) Neutron scattering function S(Q); (b) neutron PDF (c) local range of the neutron PDF, (d) neutron Bragg profile; (e) X-ray scattering function with the inset showing a magnified view of the high-Q range; (f) X-ray PDF, (g) Pb EXAFS, (h) Nb EXAFS, (i) Ti EXAFS. In (a) through (f), green lines represent the difference. In (g), (h), and (i) solid and dashed lines correspond to the magnitude and imaginary part of the Fourier transform (FT), respectively. The *k*-ranges used in the FT are 2.3 Å^{-1} to 9 Å^{-1} for Pb, 2.1 Å^{-1} to 12 Å^{-1} for Nb, and 2.2 Å^{-1} to 8.3 Å^{-1} for Ti.

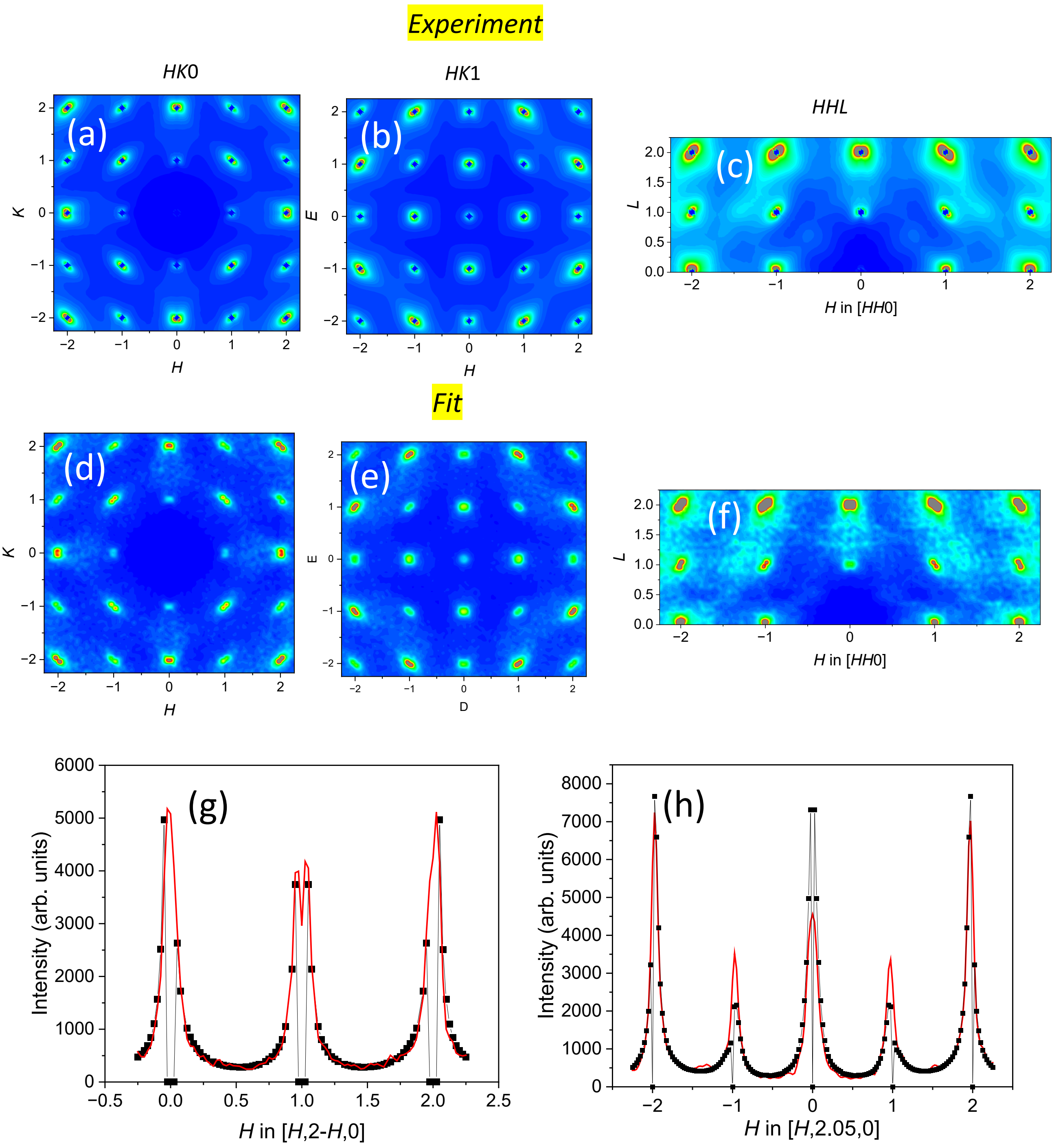

Fig. S12: **Fitting results for PMN-35PT**. *HK*0, *HK*1, and *HHL* sections of the 3D X-ray diffuse intensity distribution in PMN-35PT at 533 K fitted in RMCProfile together with the powder data shown in Fig. S11. Top row – experiment; middle row – fit; bottom row – selected traces comparing the experimental (black) and fitted (red) diffuse intensity. In the experimental data, the pixels around the saturated reflections were excluded from the fit. The procedure for calculating the diffuse scattering in RMCProfile implies that the *calculated* intensity at the exact positions of Bragg peaks is identically equal to zero. The non-zero intensity at these locations is a result of the smoothing applied to the calculated signal. This intensity is unreliable and should be ignored.

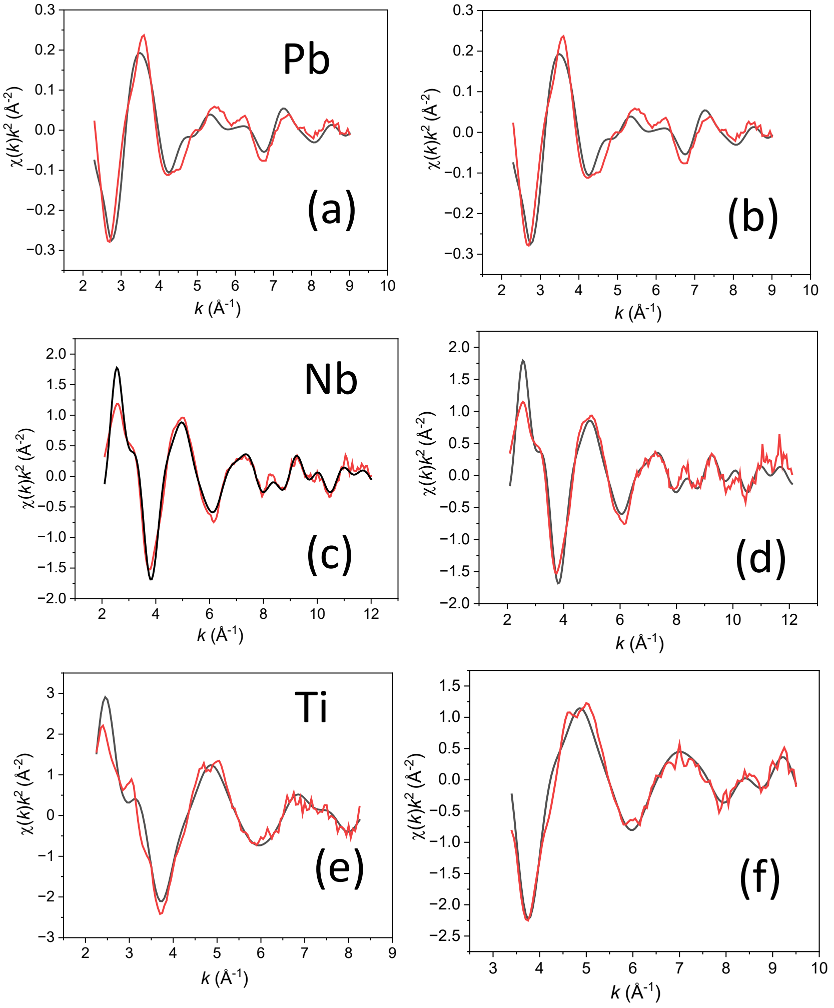

Fig. S13: **EXAFS signals in *k*-space**. Experimental (red) and calculated (black) *k*-space EXAFS signals for Pb (a, b), Nb (c, d), and Ti (e, f) in PMN-30PT (a, c, e) and PMN-35PT (b, d, f). These signals correspond to the plots of the EXAFS Fourier transforms displayed in Fig. S9 and Fig. S11. The RMC fits were performed in *r*-space. The purpose of showing the *k*-space signals is to illustrate the discrepancy between the experimental and calculated signals relative to the noise levels in the experimental data, which in the *r*-space are masked by a smoothing effect of the Fourier transform.

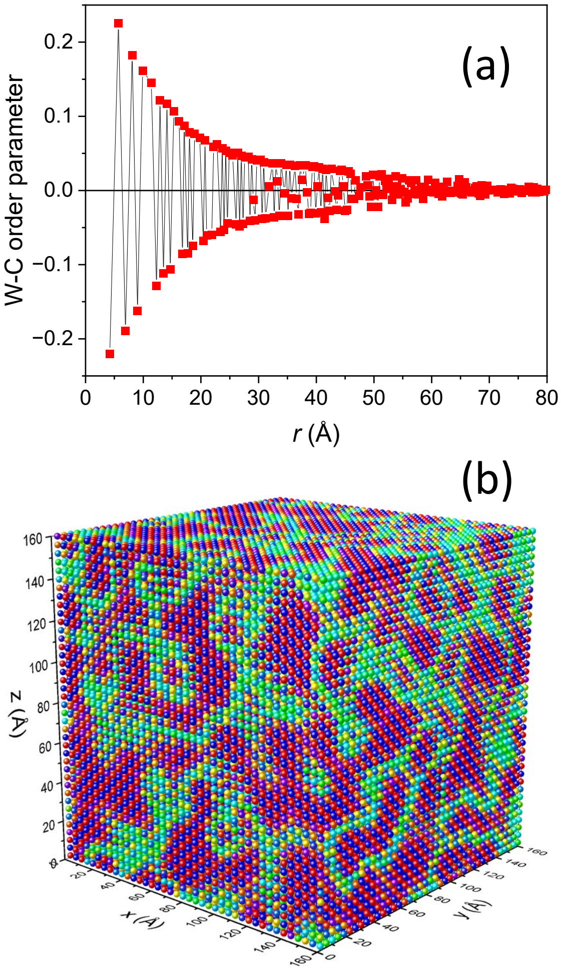

Fig. S14: **B-cation ordering in PMN**. (a) Warren-Cowley order parameter calculated for the octahedral sites occupied by Nb and Mg is shown as a function of interatomic distance. Black line is a guide to the eye. The change in the sign of this parameter for successive coordination shells is consistent with the rocksalt-type ordering of the two species. (b) 3D rendering of B-cations in the PMN configuration with color representing the intensity of the inverse Fourier transform (IFT)of the calculated amplitude for the ½111-type diffuse superlattice reflections. This Fourier filtering highlights nanoscale regions with the rocksalt-type ordering of Mg and Nb into alternating {111} planes (red and blue). Green-colored regions acquire weak intensity of the IFT, signifying weaker albeit non-zero ordering, consistent with [10].

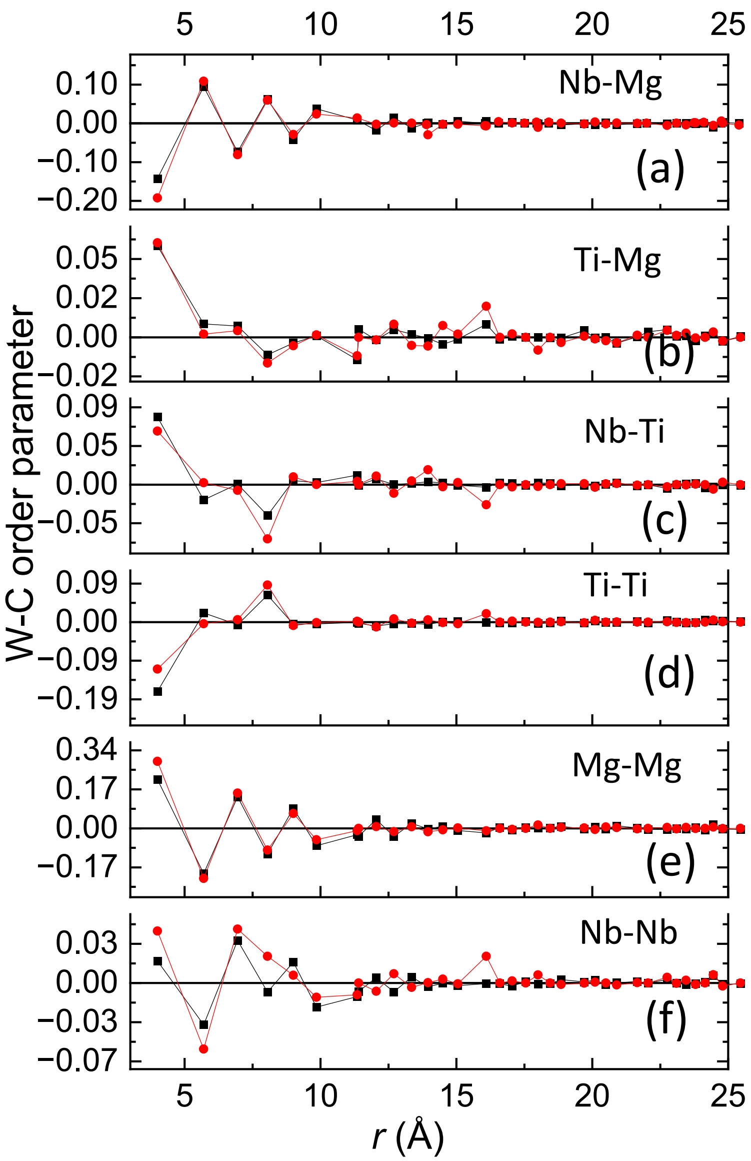

Fig. S15: **Warren-Cowley order parameters in PMN-PT**. The parameters are shown for the distinct pairs of octahedral cations as a function of interatomic distance. Red - PMN-30PT; Black - PMN-35PT. (a) Nb-Mg; (b) Ti-Mg; (c) Nb-Ti; (d) Ti-Ti; (e) Mg-Mg; (f) Nb-Nb. The behavior for Nb and Mg is consistent with their rocksalt-type ordering as in PMN but limited to much shorter distances of about 1 nm. For the pairs involving Ti, no clear ordering pattern can be inferred. The values of the order parameters for the PMN-PT compositions should be treated only as approximate estimates because of the inherently limited number and weak intensity of the superlattice reflections available for the analysis.

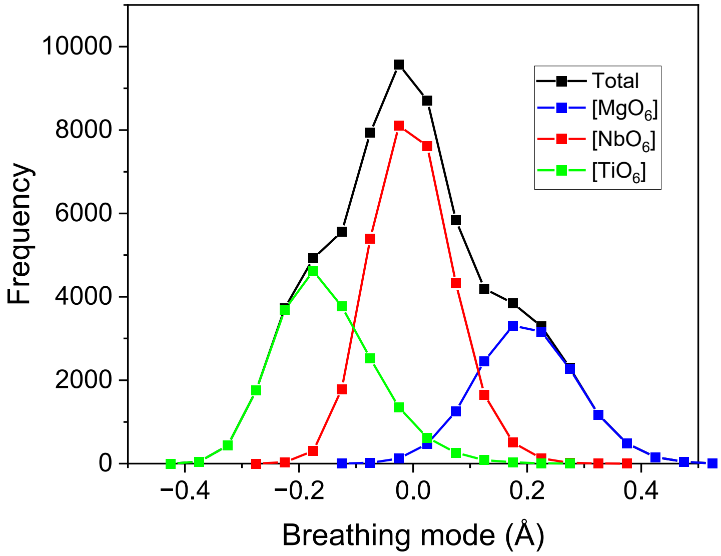

Fig. S16: **Chemically-resolved octahedral size in PMN-30PT**. Statistical distribution of the breathing-mode magnitude in PMN-30PT which reflects oxygen displacements that yield a uniform expansion (positive) or contraction (negative) of octahedral volumes relative to the average.

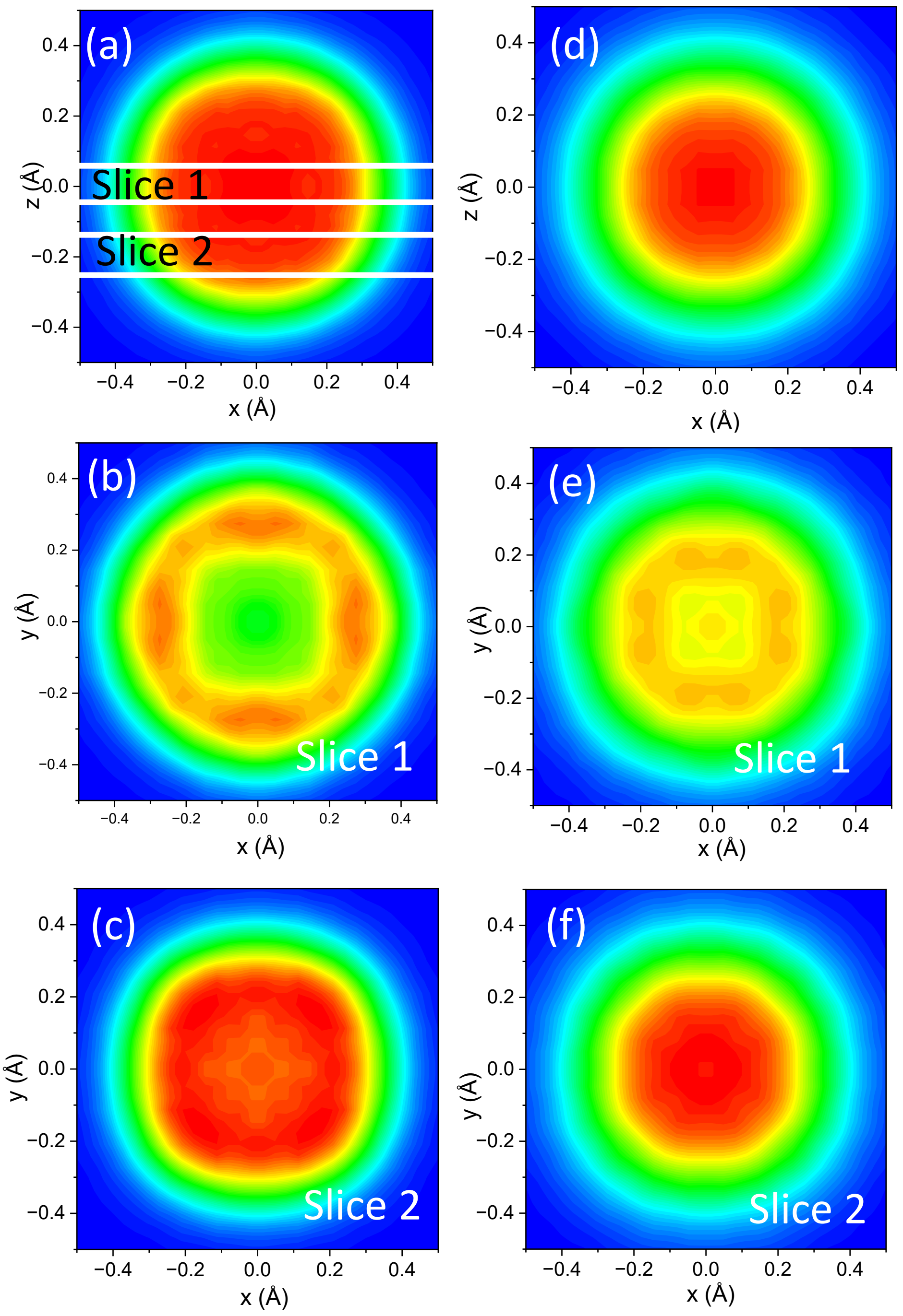

Fig. S17: **Pb probability densities distributions**. Projected probability density distributions (PDD) for Pb in PMN at 300 K (a-c) and 490 K (d-f). (a) and (d) represent the symmetrized projections on the (001) plane. (b) and (c) display slices 1 and 2 through the distribution in (a) while (e) and (f) show similar slices for (d). At both temperatures, the Pb PPDs exhibit minima at the average {0,0,0} 1*a* Wyckoff position. The Pb atoms are offset preferentially (≈10 % higher probability compared to the random case) along ⟨111⟩ and ⟨100⟩ directions. The preference for ⟨111⟩ displacements is most pronounced, in agreement with the previous refinements using the same powder data (see Methods section).

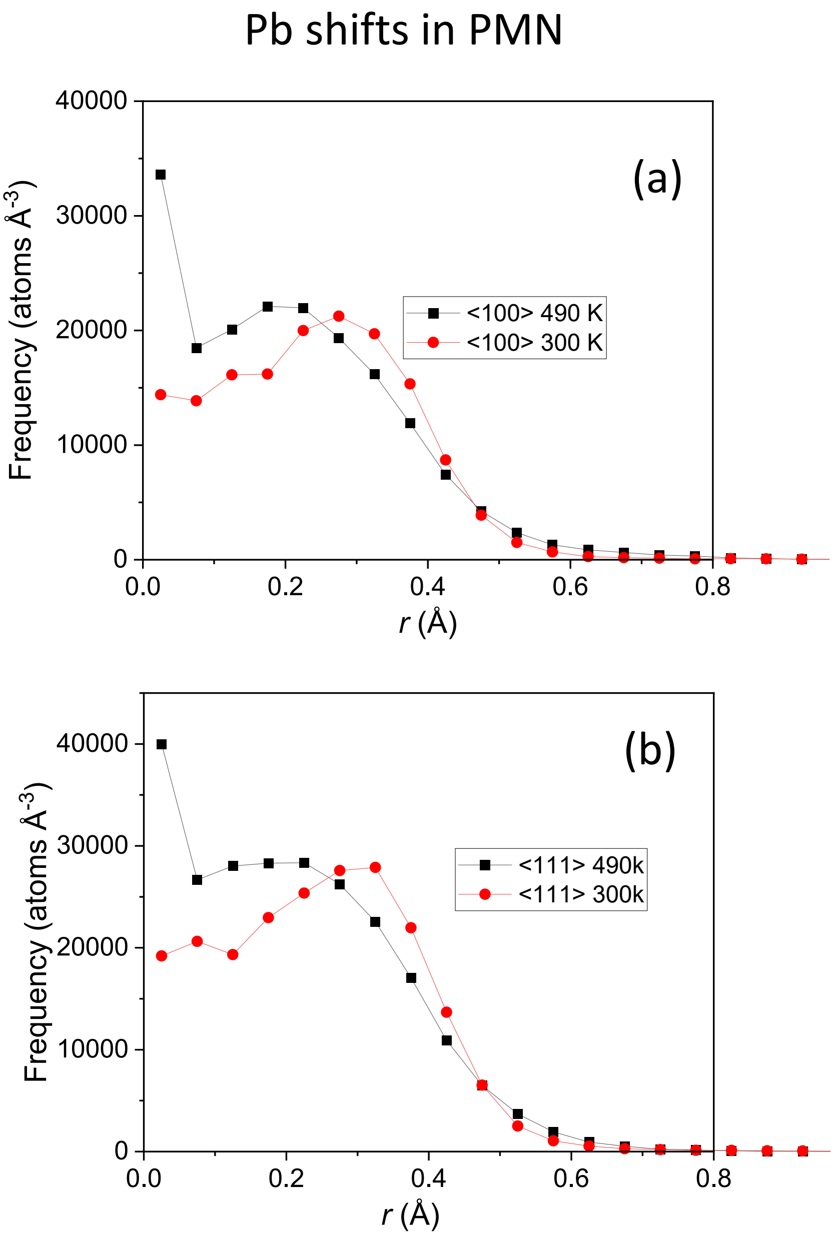

Fig. S18: **Distributions of Pb displacements**. Statistical distributions of the magnitudes of Pb displacements in PMN at 300 K (red) and 490 K (blue) along the ⟨100⟩ (a) and ⟨111⟩ directions (b). The offset of Pb from the average position increases by ≈0.1 Å on cooling from 490 K (≈0.2 Å) to 300 K (≈0.3 Å), consistent with MD simulations [14] attributed this increase to the growth of static components of the displacements, with the distributions of displacements becoming narrower at 300 K.

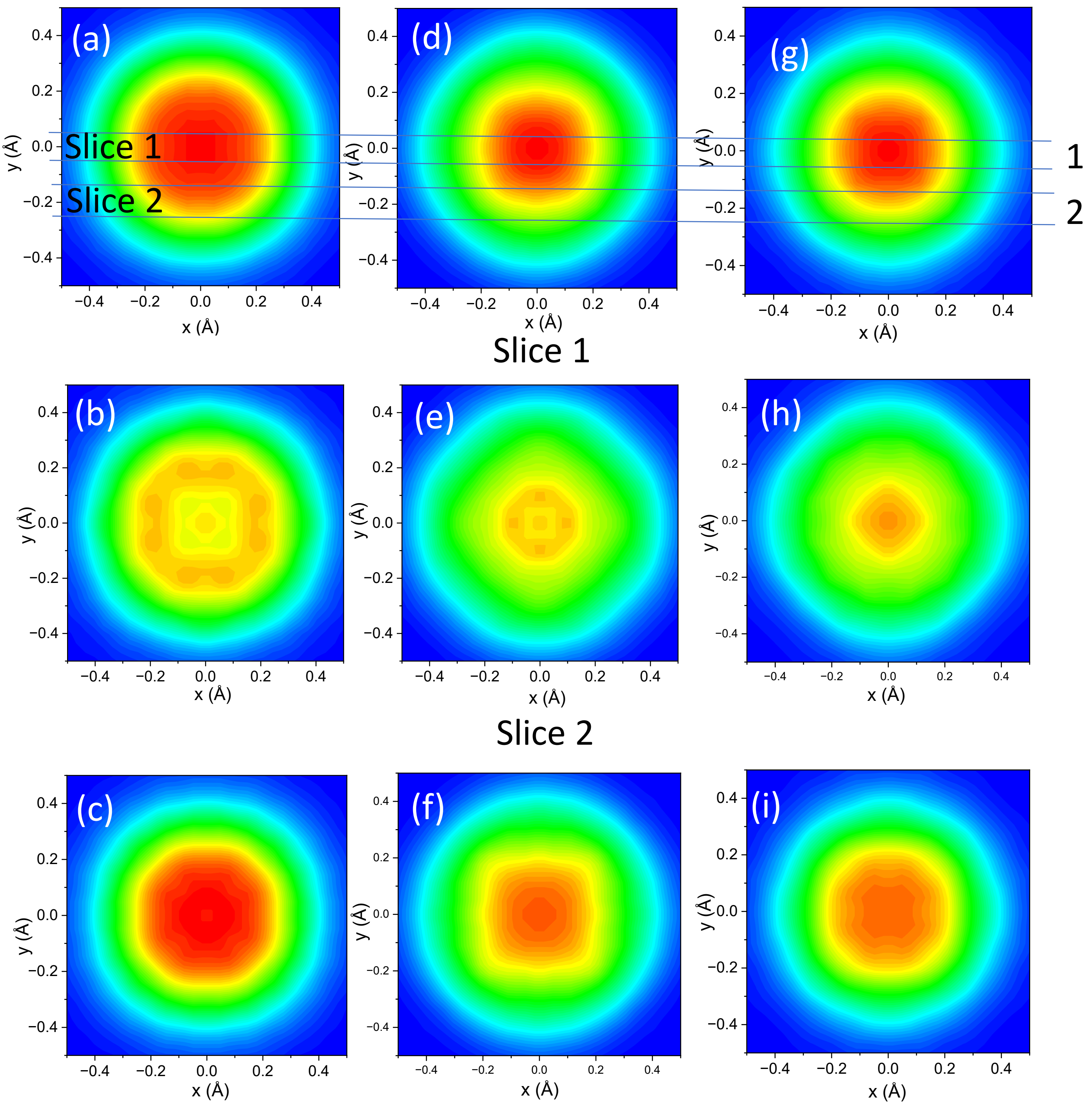

Fig. S19: **Pb probability density distributions**. Comparison of the Pb probability density distributions for PMN at 490 K (a, b, c) with PMN-30PT (d, e, f) and PMN-35PT (g, h, i) at 533 K. For the PMN-PT compositions at 533 K, Pb PDDs are visibly tighter than in PMN at 490 K, and the probability dip in the center, while still observable for PMN-30PT, disappears for PMN-35PT. The directional preference for the Pb displacements changes to ⟨100⟩ with the ⟨111⟩ shifts becoming secondary. The probability of the latter is still larger than random for PMN-30PT, but this difference almost vanishes for PMN-35PT. The anisotropy of the corresponding Pb PDDs changes accordingly, with an increasingly close overlap of the four ⟨100⟩ off-centered probability maxima in the central section. A smaller magnitude of the Pb off-centering for the PMN-PT compositions compared to pure PMN can be attributed to the overall progressive reduction in the lattice volume upon substituting Ti which has a smaller ionic radii than Mg and Nb.

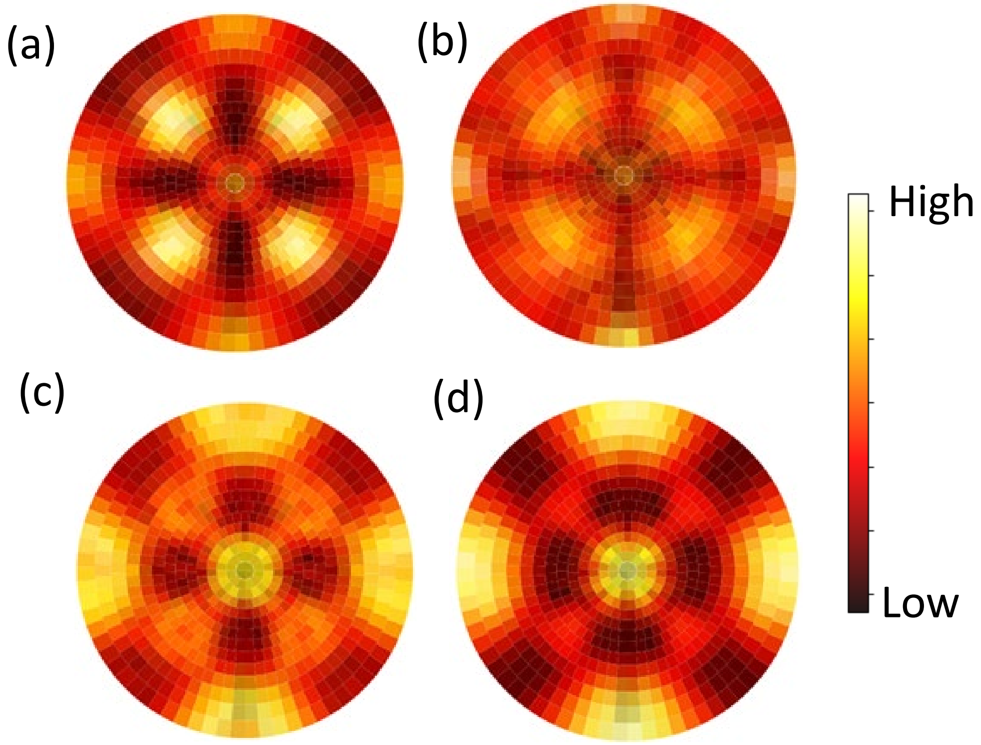

Fig. S20: **Directions of Pb displacements**. Stereographic projection of symmetrized probability-density maps for the directions of Pb displacements off the ideal cubic position in (a) PMN at 300 K, (b) PMN at 490 K, (c) PMN-30PT at 533 K, and (d) PMN-35PT at 533 K. Color reflects the fraction of atoms per pixel.

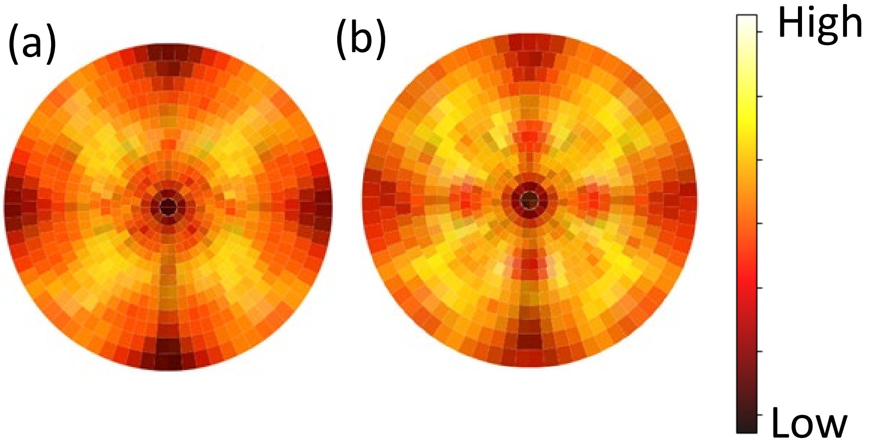

Fig. S21: **Directions of Nb and Mg displacements**. Stereographic projections of symmetrized probability-density maps for the directions of (a) Nb and (b) Mg displacements off the ideal cubic position in PMN at 300 K. Both cation species exhibit a preference for displacements along ⟨111⟩ directions. Color represents the fraction of atoms per pixel.

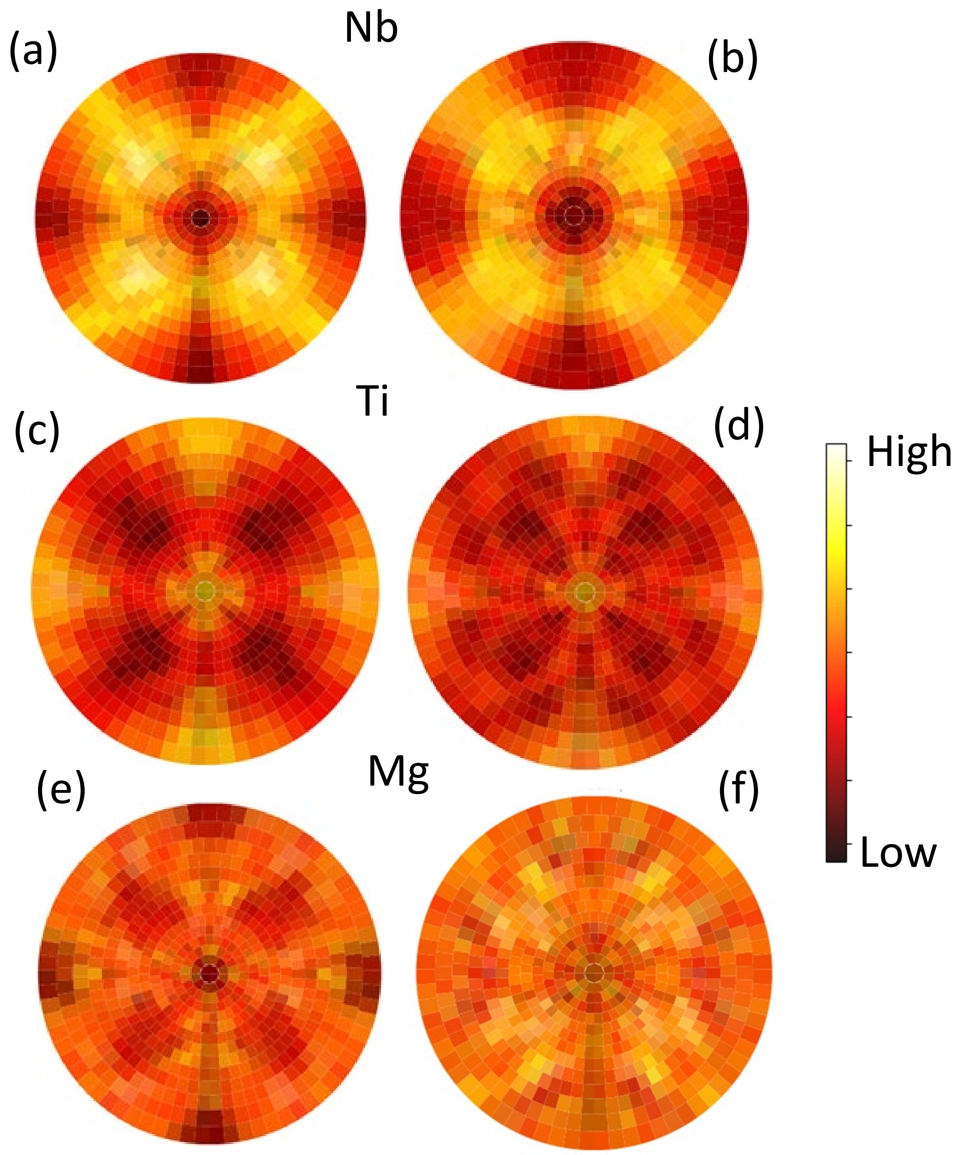

Fig. S22: **Directions of Nb, Ti, and Mg displacements**. Stereographic projections of symmetrized probability-density maps for the directions of Nb (a, b), Ti (c, d), and Mg (e, f) displacements off their ideal cubic position in PMN-30PT (a, d, e) and PMN-35PT (b, d, f). Nb exhibits a clear preference for the displacements along ⟨111⟩, whereas Ti cations are preferentially shifted along ⟨100⟩ directions. For Mg, no such clear preferences are evident. Color represents the fraction of atoms per pixel.,

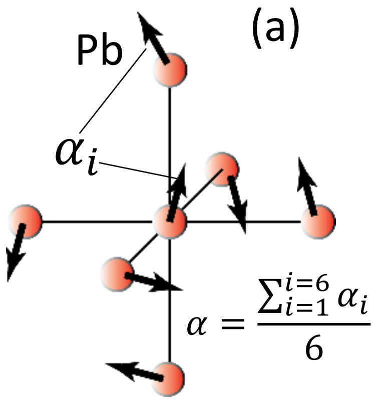

Fig. S23: **Metric for the local alignment of displacements**. Schematic drawing of a $[PbPb_6]$ cluster with the Pb atoms and their displacement vectors represented using spheres and arrows, respectively. α_i denotes an angle between the displacement vectors for the central and i^{th} neighboring atom. The average angle α for this cluster is calculated using the formula in the figure. A similar definition α was used for the $[PbB_8]$ and $[BPb_8]$ coordination environments.

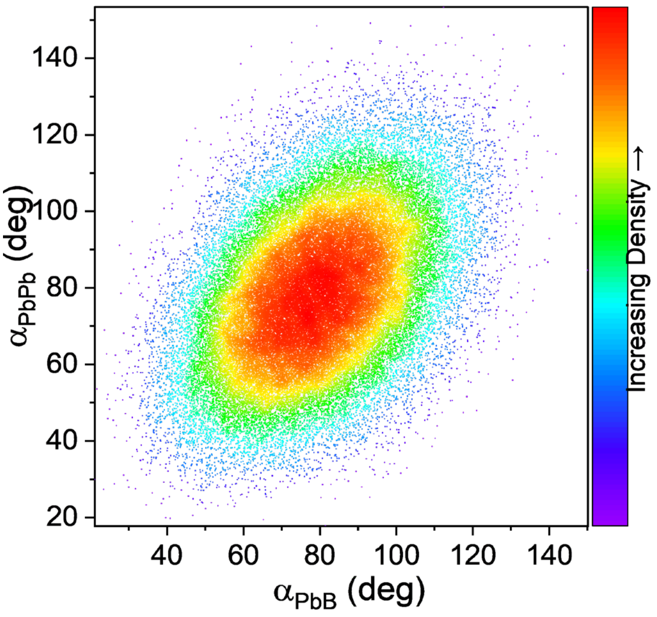

Fig. S24: **Alignment of near neighbor displacements**. Angle α within the [$PbPb_6$] and[$Pb(Nb,Mg)_8$] clusters. A strong correlation between the two is evident.

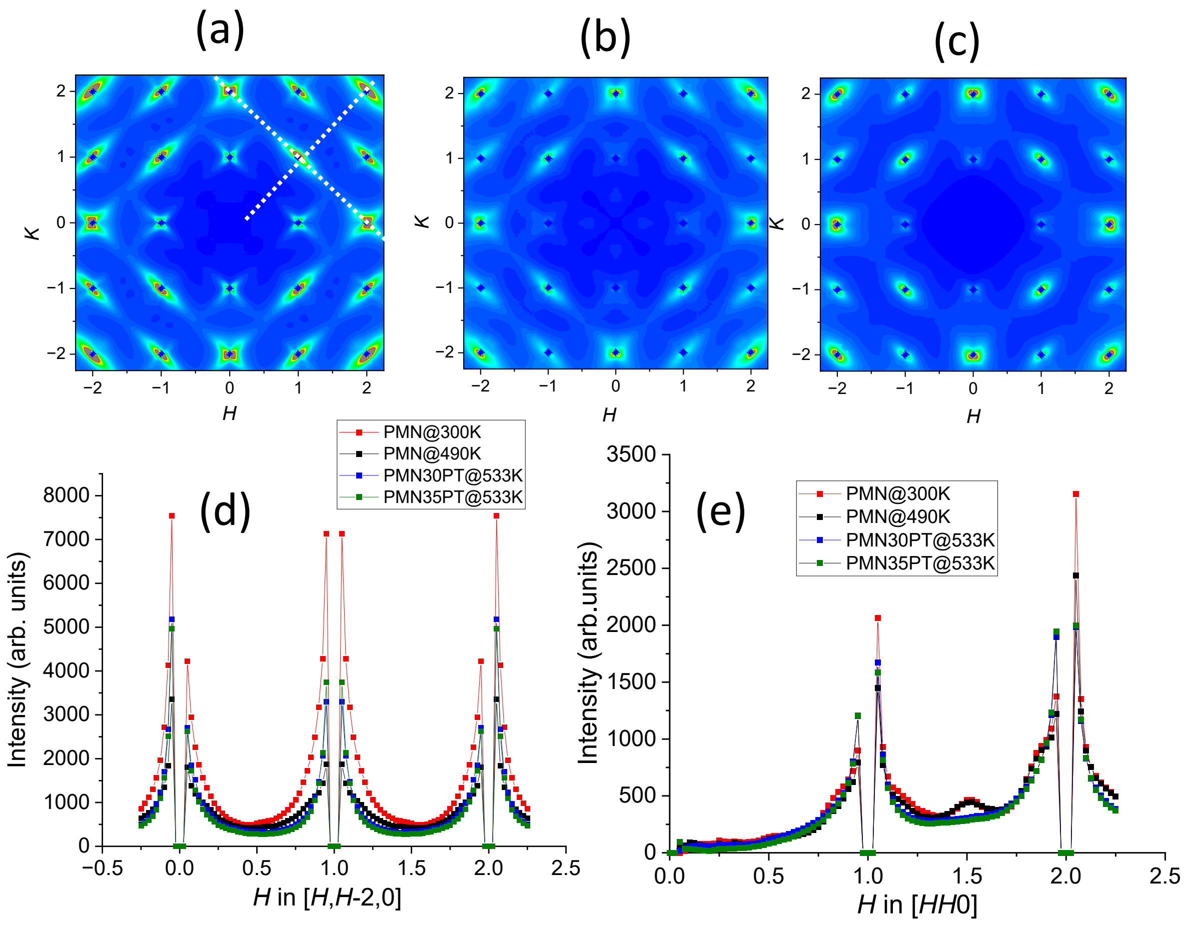

Fig. S25: **Comparison of diffuse intensity distributions**. *HK0* sections of the 3D X-ray diffuse-scattering datasets in (a) PMN at 300 K, (b) PMN at 490 K, (c) PMN-30PT at 533 K. (d) Intensity traces along the [*H*,*H-2*,0] directions for PMN at 300 K and 490 K, PMN-30PT, and PMN-35PT. (e) intensity traces along the *HH*0 direction. Dashed lines in (a) indicate the directions for the traces. The weak peak at *H*=1.5 in (e) represents the intensity distribution across the tail of the diffuse scattering extending along the orthogonal direction. This peak is similar for PMN at 300 K and 490 K (apart from extra broadening at 490 K) but absent for both PMN-PT compositions.

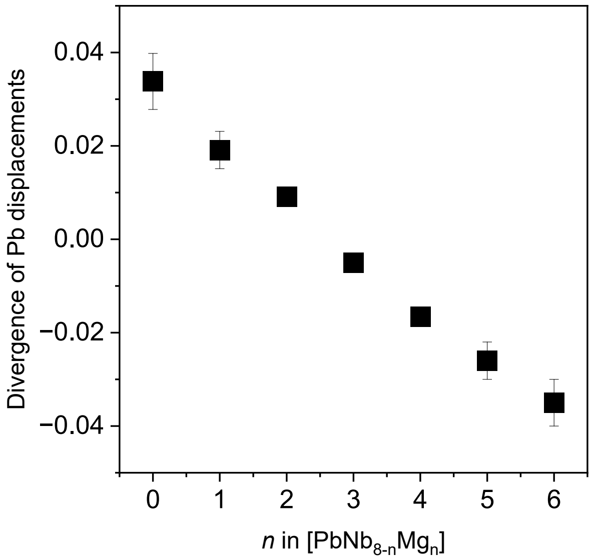

Fig. S26: **Divergence of the Pb displacement field as a function of composition**. The divergence metric (see Methods) was calculated for the correlated components of Pb displacements in PMN at 300 K as a function of the local Mg content around Pb. Error bars reflect a single standard deviation estimated from the analysis of three refined configurations. The sign of the divergence changes from positive for the Nb-rich environments to negative for Mg-rich.

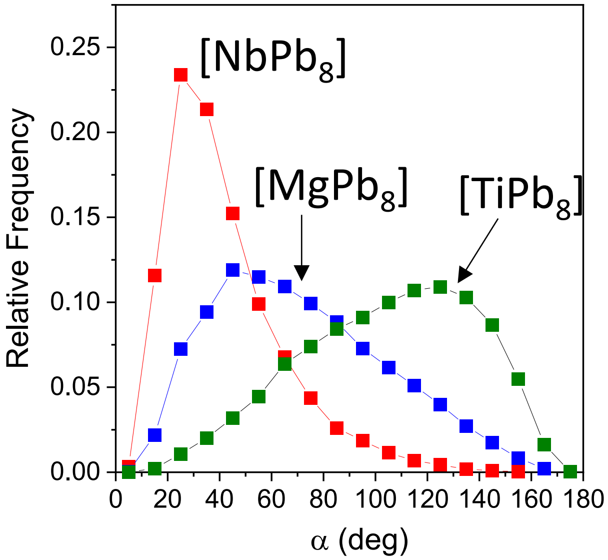

Fig. S27: **Alignment of B-cation and Pb displacements**. Statistical distributions of the alignment angle α calculated for the B cations and their neighboring Pb atoms in PMN-30PT. The distributions indicate a clear preference for the parallel alignment between the Nb and Pb displacements and antiparallel between those of Ti and Pb.

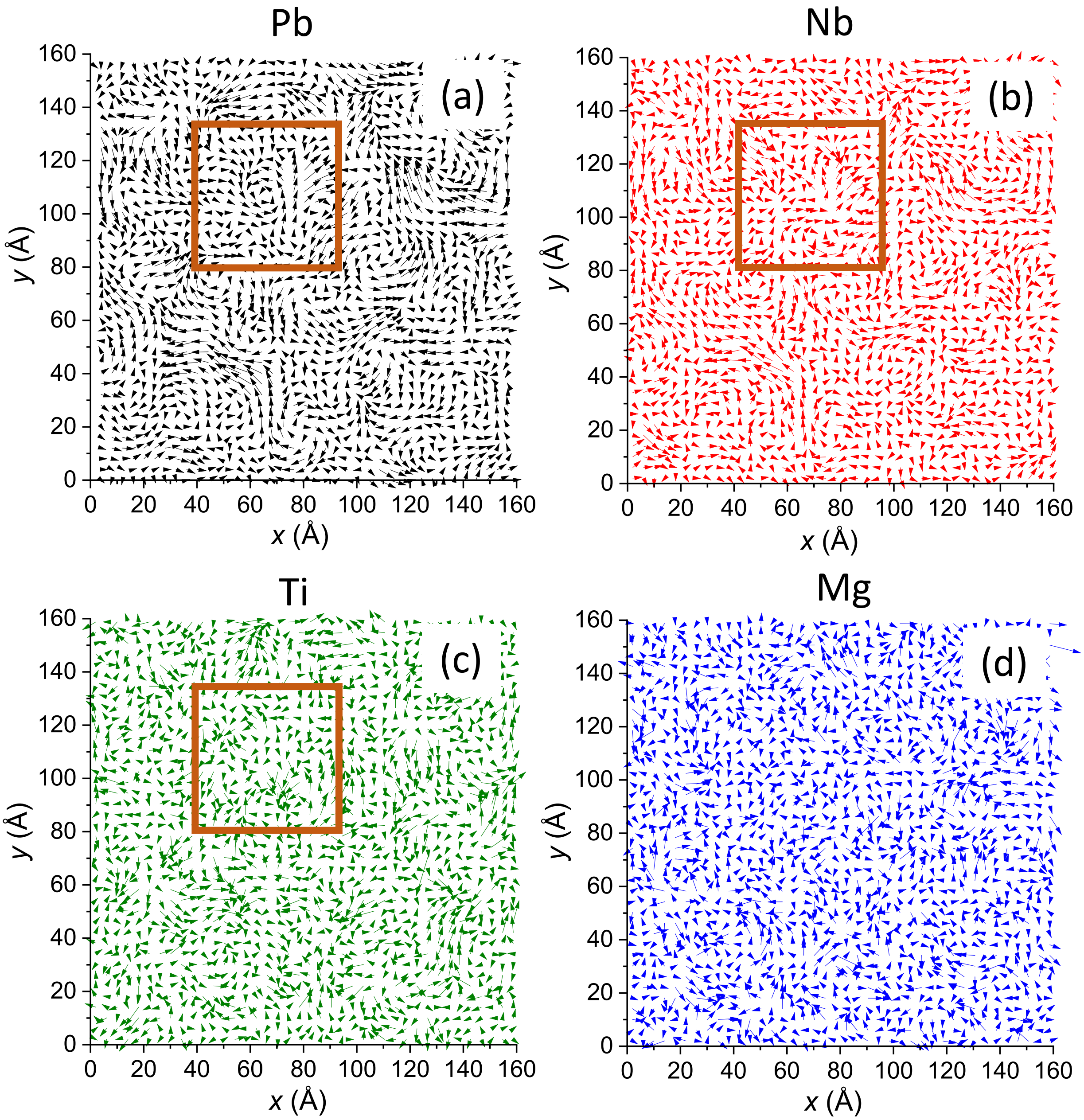

Fig. S28: **Displacement maps in PMN-30PT**. Maps of displacements for the cation columns in PMN-30PT projected onto the *x-y* plane. (a) Pb; (b) Nb; (c) Ti; (d) Mg. Atomic coordinates in the 3D configurations used to calculate these projections were obtained as sums of the corresponding average coordinates and correlated components of displacements determined from the Fourier filtering of the calculated diffuse scattering amplitude. The column displacements for Pb and Nb are positively correlated, whereas those for Pb and Ti exhibit negative correlations, as can be seen from the comparison of the patterns in the area outlined using a square. The displacements of Mg columns also exhibit some correlations but their patterns are less obvious.

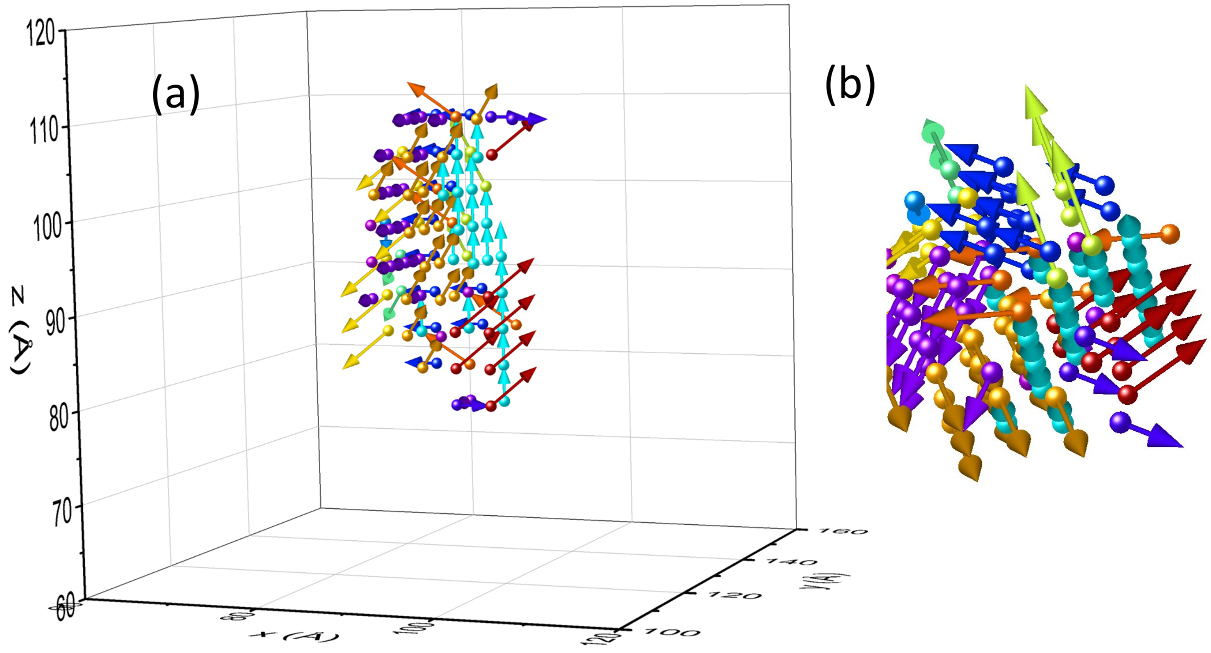

Fig. S29: **Clustering of Pb displacements within a meron in PMN**. (a) Cluster of Pb atoms forming a meron-like defect in the refined configuration of PMN at 300 K. The arrows represent displacement directions. Their color corresponds to one of the ⟨111⟩ and ⟨100⟩ directions falling within the 35° tolerance angle from the displacement vector. The meron consists of 71° pairs of ⟨111⟩ variants and 54° pairs of ⟨111⟩ and ⟨100⟩ variants. (b) Magnified view of the same cluster along the meron axis.

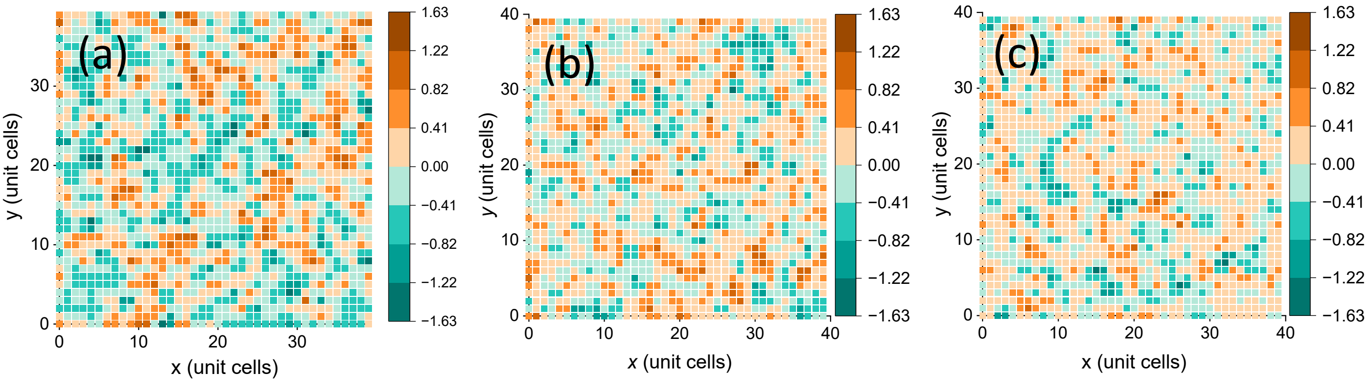

Fig. S30: **Electric charge distribution.** Maps of electric charge calculated in a single layer of unit cells in In the refined configurations of (a) PMN, (b) PMN-30PT, and (c) PMN-35PT. The calculations assumed formal ionic charges.

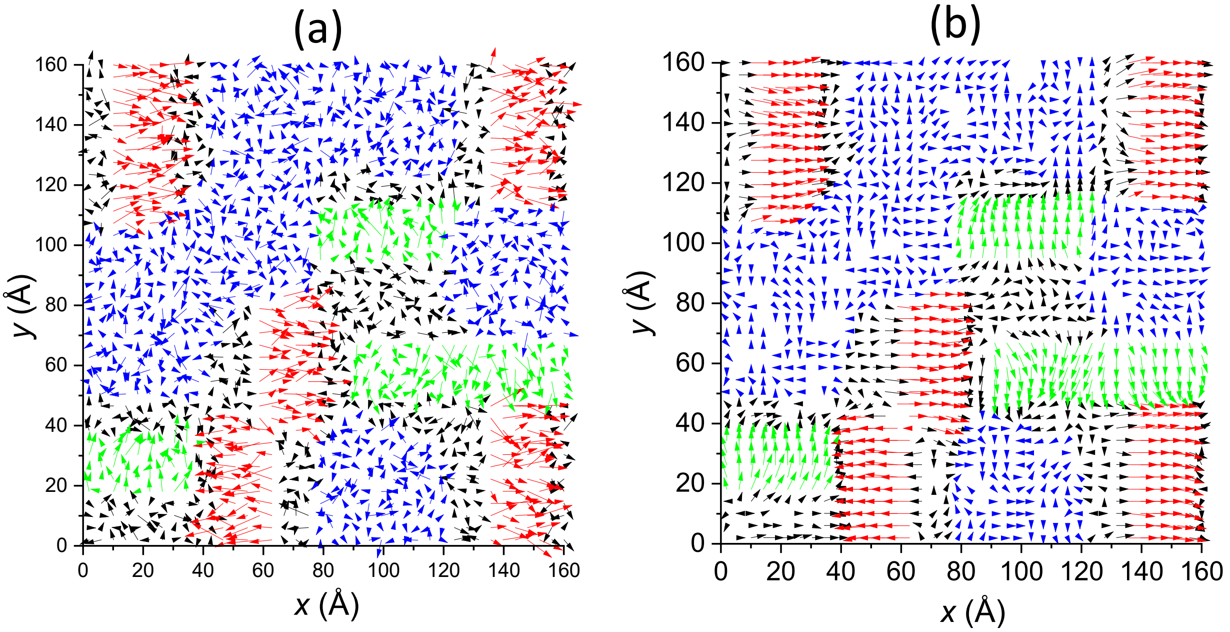

Fig. S31: **Effect of the Fourier filtering**: (a) Atomic layer parallel to the *x*-*y* plane in a simulated configuration that contains three orientational domain variants with the displacements parallel to the *x* (red), *y* (green), and *z* (blue, out of plane) directions. The configuration also contains a small volume fraction of a matrix with disordered displacements (black). All the atoms were additionally displaced according to a Gaussian distribution to imitate thermal disorder. (b) Same layer but with displacements filtered using the Fourier transform of the diffuse scattering amplitude around the Bragg reflections calculated for the simulated configuration. Evidently, the filtering removes the random component emphasizing the correlated components in each domain.

Table S1: Components of the Warren-Cowley order parameter for the first cation-cation coordination sphere in *x*=0.3. The standard deviation of these values estimated from several independently refined atomic configuration is estimated as ±0.05.

Species	Mg	Ti	Nb
Mg	0.21	0.05	-0.14
Nb	0.05	-0.17	0.08
Ti	-0.14	0.08	0.019

Table S2: Components of the Warren-Cowley order parameter for the first cation-cation coordination sphere in *x*=0.35. The standard deviation of these values estimated from several independently refined atomic configuration is estimated as ±0.05.

Species	Mg	Ti	Nb
Mg	0.21	0.05	-0.14
Nb	0.05	-0.17	0.08
Ti	-0.14	0.08	0.019
